# Conflicts in Romantic Relationships over Facebook Use: Validation and Psychometric Study

**DOI:** 10.3390/bs9020018

**Published:** 2019-02-10

**Authors:** Juan Aníbal González-Rivera, Idania Hernández-Gato

**Affiliations:** 1 School of Behavioral and Brain Sciences, Ponce Health Sciences University, San Juan University Center, 388 Zona Industrial Reparada 2, Ponce, PR 00716, USA; 2 San Juan Campus, Carlos Albizu University, 151 Calle Tanca San Juan, PR 00901, USA; ihernandez759@sunmail.albizu.edu

**Keywords:** Facebook, Facebook intrusion, couple relationships, conflicts, jealousy, psychometric properties, validation

## Abstract

The present study evaluates the psychometric properties of the Conflicts in Romantic Relationships Over Facebook Use Scale with a sample of Puerto Rican adults. A total of 300 Puerto Ricans participated in this confirmatory and psychometric study. The results confirmed that the scale has a multidimensional structure. These dimensions are: Partner Facebook intrusion, Conflict over Facebook use, and Jealousy over Facebook use. A total of 18 items complied with the criteria of discrimination and presented appropriate factorial loads (six items per dimension). The Cronbach’s alpha indexes of the dimensions ranged between 0.87–0.95, and the omega coefficients ranged between 0.88–0.95. In summary, the instrument has the appropriate psychometric properties to continue with validation studies, as well as to be implemented in various work areas, both theoretical and applied.

## 1. Introduction

Facebook (FB) is considered the most popular social network site (SNS). At the end of third quarter of 2018, the platform had 2.27 billion monthly active users worldwide [1]. For the same year, statistics regarding daily use indicated that more than half of United States residents (53%) use FB several times a day, average access to FB is eight times per day, and 35 million users update their statuses daily [2]. This SNS has several implications on its users’ interpersonal life, given the opportunities that they encounter to establish new relationships and maintain current ones [3]. However, despite the current advantages that SNSs provide, some authors sustain the negative effects associated with their continuous use [4,5,6]. For example, regarding romantic relationships, excessive attachment to FB might generate conflicts, disagreements, discussions, and jealousy in the relationship [5,6,7,8]. 

Even though in other countries there are instruments that measure variables associated with conflicts in romantic relationships related to technology [9] and cell phone use [10], neither in Latin America nor Puerto Rico are there instruments in Spanish that measure the consequences of the excessive FB use in romantic relationships. Considering this, the objective of this study was to develop a scale that allows the measurement of conflicts over FB use in a tridimensional model (Partner FB intrusion, Conflict over FB use, and Jealousy over FB use). The creation of a valid and reliable instrument that explores conflicts in romantic relationships due to FB intrusion will be of added value to the scientific community that explores these issues, mainly in Latin America and the Caribbean.

### 1.1. Theoretical Framework

Romantic relationships could be defined as the free and voluntary union of two individuals who share a life project of common existence that is long-lasting, in which strong feelings of belonging are generated; there is a personal commitment among its members, and intense linkages of intimacy, reciprocity, and dependence are established [11]. In this regard, it is timely to make explicit that this definition includes both heterosexual and homosexual couples, couples who have formalized their relationship legally through marriage and those who have not, as well as cohabiting couples and couples that are not living together. Whereas, conflict is defined as a social phenomenon that occurs in the interaction among individuals or groups, where there are disagreements and incompatibilities, and hostilities may develop. In terms of romantic relationships, response patterns to conflicts and stress coping styles have implications in the future of the relationship [12].

There are several theoretical models that explore stress in romantic relationships, among them is the systemic transactional model of dyadic coping [13,14]. This model is focused on the impact of the effect of daily stress in relationship functioning (time share together, communication, and well-being), and how those mediators are associated with relationship satisfaction and the probability of breakup. The model suggests that everyday stressors affect relationship functioning, causing disaffection, and slowly deteriorating its quality over time. Specifically, the model proposed that stress will affect the quality of the romantic relationship as follows: (a) decreasing the shared time, considerably affecting their emotional intimacy; (b) decreasing the quality of communication; (c) increasing the risk of physical and psychological problems; and (d) increasing the likelihood that the most problematic features of personality will be expressed between the partners in the form of rigidity, anxiety, and hostility. The likelihood of distance and separation increases when partners talk less about their private experiences, needs, and interests, which gradually favors the presence of more conflicts [14].

The development of information and communications technologies have favored the use of the internet and social networks to become one of the stressors in romantic relationships. Scientific studies have been consistent in demonstrating that the intrusion or interference of technology in interpersonal relationships can generate intrafamily conflicts, as well as negatively affect romantic relationships [9,10]. There is even scientific literature that suggests that the stress caused by the interference of technology and social networks deteriorates the general well-being of individuals [9,10,15].

### 1.2. Facebook Intrusion in Romantic Relationships 

FB intrusion is characterized by an individual’s constant need to access FB, which interferes with its daily functioning, and as a result, interpersonal relationships are impacted [6]. Studies that explore FB intrusion and its consequences on the diverse aspects of an individual’s life are currently limited. Some research has associated this intrusion with variables such as depression [16,17,18], low self-esteem [4,18], fear of rejection [18], and an intense need to be accepted by others [4]. Likewise, individuals with high levels of FB intrusion can experiment distress in moments where they are unable to access FB [6], and they usually show characteristics that are frequently observed in people with addiction disorders, such as tolerance, withdrawal, and relapse [19]. 

As mentioned above, the constant FB use can cause a negative effect on interpersonal relationships, especially romantic relationships. This happens because the deep emotional attachment to this SNS interferes with the couple’s daily activities. Consequently, the members of the relationship may feel tense, insecure, and unsatisfied [6]. Likewise, other authors have pointed out the negative impact of addictive behaviors on intimate relationships and emphasized the lack of satisfaction among the members of the relationship when FB becomes a nuisance when intervening within daily relationship functioning [8]. In this research, we will use the term Partner FB intrusion to refer to how a person perceives that the use of FB by their partner interferes or interrupts the physical and emotional interaction that they may have, such as accessing FB when they eat, talk, or share time together.

### 1.3. Conflicts over Facebook Use

It has been shown in several studies that the use of technology may cause conflicts in romantic relationships, negatively affect the communication between couples, and impact the emotional well-being of the members at times when they are sharing quality time together [9,10,20]. Alike, the way in which one of the members perceives technology use by its partner plays an important role in relationship satisfaction [9,21]. Another study demonstrated that relationship intimacy is affected by the partner’s perception toward FB use, and not only by using the SNS [22]. Identifying the use of FB as a problematic issue creates a barrier that weakens couple’s intimacy and, as a result, significant conflicts can be developed. 

Internet use in general, as well as the excessive use of SNSs (e.g., FB), has shown to be a threat against romantic relationships [5]. Some people use FB to monitor their partner’s activities. It has been proven that these behaviors are highly counterproductive, since they tend to create conflicts in the relationship, and be a possible precursor for future breakups [23]. For example, a study conducted with 190 newlyweds revealed that compulsive Internet use deteriorates the relationship and causes negative feelings in the affected partner [24]. On the other hand, a research study conducted with South Asian, Europeans, and North American participants confirmed the negative effects of the excessive FB use when reaffirming that those behaviors result in the decrease of relationship quality [25]. 

### 1.4. Jealousy over Facebook Use

Despite the positive effects, such as feelings of satisfaction and social integration, that various researches confirm over FB use [26,27,28], other studies have suggested that excessive behaviors (e.g., spend much of the time in social networks) can predispose jealousy in romantic relationships, and as a result, relationship satisfaction may decrease. A research conducted in Australia was the first exploring the impact of FB intrusion and jealousy in relationship satisfaction [6]. The findings confirmed that relationship satisfaction is only affected negatively when FB intrusion generates jealousy and one of the members in the relationship engages in surveillance behaviors. 

Another research conducted in Canada, with a sample composed mostly of women between the ages of 17–24 years, revealed a significant association between the time spent on FB and jealousy as a response to this behavior [8]. In this study, participants expressed the feelings of insecurity caused by FB. Even participants who had full confidence in their partners became jealous in situations where other people posted messages on their FB wall. Some expressed that they understood that their feelings of jealousy could be real or imaginary, and those who already felt jealousy and insecurity in their relationship expressed that FB had worsened the situation. In this study, as well as in other research, women obtained higher scores on jealousy compared to men [7,8]. 

### 1.5. Instruments to Measure Conflicts in Romantic Relationships

Some researchers have made efforts to validate instruments that allow the measurement of variables associated with conflicts in romantic relationships due to the use of technology. For example, Elphinston and Noller [6] contributed to the advancement of this field through developing the Facebook Intrusion Questionnaire (FIQ), which consists of eight items and obtained an internal consistency index of 0.85. The FIQ allows a self-evaluation of the cognitive and behavioral areas related to FB use, possible conflicts, as well as other consequences, such as the emergence of certain behaviors observed in people with addiction disorders. It should be mentioned that the FIQ was not designed to assess Partner FB intrusion. There are other instruments that do not directly measure FB intrusion, but they evaluate the interference of technology in romantic relationships. One of them is the Partner Phubbing Scale [10], which consists of nine items that measure how a person perceives that his/her partner ignores him/her in favor of paying more attention to their mobile device. González-Rivera, Segura, and Urbistondo [29] translated and validated the scale in a sample of Puerto Rican adults, obtaining outstanding psychometric properties and an adequate internal consistency index (α = 0.93). Other measurements available include the Technology Device Interference Scale (TDIS) and the Technology Interference in Life Examples Scale (TILES), which enable assessing the interference of technology and the participant’s perception about this interference in their romantic relationship. Both scales were developed by McDaniel and Coyne [9], and they obtained an internal consistency index of 0.67 and 0.85, respectively. 

Regarding the instruments that assess conflicts in romantic relationships, there is a questionnaire developed by Clayton, Nagurney, and Smith [5] that measured the negative effects in romantic relationships as a result of FB use. The questionnaire obtained an internal consistency index of 0.85. On the other hand, the Conflict over Technology Use Scale [9] evaluates the frequency with which participants perceives that technology causes conflicts in their relationship. At the same time, Roberts and David [10] developed the Cell Phone Conflict Scale, which consists of 10 items that measure a participant’s perception related to cell phone use, as a source for the development of conflicts in their romantic relationship. This scale was translated and validated by González-Rivera et al. [29], showing an appropriate internal consistency index of 0.91. 

As for jealousy, Muise, Christofides, and Desmarais [8] developed the Facebook Jealousy Scale, which compiled a list of items that displays the aspects of this SNS that have the potential to be a trigger for romantic jealousy. The scale has 27 items and an internal consistency index of 0.96. In summary, there is no instrument in Spanish or English that simultaneously evaluates partner FB intrusion, conflicts associated with this behavior, and the jealousy created in response.

### 1.6. Purpose of the Study

The objective of this study is to develop, validate, and examine the psychometric properties of the Conflicts in Romantic Relationships Over FB Use Scale using advanced statistics. Explicitly, this study has four main objectives:

1. Analyze the factor structure of the Conflicts in Romantic Relationships Over FB Use Scale through confirmatory factor analysis with structural equations.
2. Analyze the discrimination capacity of the instrument’s items.
3. Analyze the reliability of the instrument and its factors through the internal consistency indexes of Cronbach and omega.
4. Analyze the convergent and divergent validity of the factors through the analysis of average variance extracted (AVE), maximum shared variance (MSV), and the average shared variance (ASV).

## 2. Methods

### 2.1. Research Design

This study has an instrumental design [30], where all of the psychometric properties of the Conflicts in Romantic Relationships Over FB Use Scale through confirmatory factor analysis were examined. In this way, the factor structure of the instrument was tested, and the proposed objectives were met. This research was approved by the Institutional Ethics for Research Committee of the Carlos Albizu University, San Juan Campus, Puerto Rico (Code SP18-32). The data compilation was carried out by using online questionnaires through the PsychData platform and posting a paid ad in the main social networks as a recruitment method: FB, Twitter, Google+, and WhatsApp, among others. This ad redirected the participants to the online survey, where they read the informed consent, which notified them of the following: (a) the purpose of the study, (b) inclusion criteria, (c) the voluntary nature of the study, (d) possible risks and benefits, and (e) their right to withdraw from the study at any time. To guarantee the privacy and confidentiality of the participants, the questionnaires were completed anonymously, and they were able to print a copy of the informed consent. 

### 2.2. Participants

A non-probabilistic sample of 300 adults, with an average age of 32.87 (SD = 7.096) was used. Sociodemographic data of the sample is presented in Table 1. The following inclusive criteria was established for participating in the study: (1) to be of 21 years or more, (2) be a Puerto Rican resident, (3) be in a relationship for one year or more (married or cohabiting), and (4) partner must have an active FB account. 

### 2.3. Measurement

Sociodemographic Data. To identify the sociodemographic characteristics of the sample, we developed a general data questionnaire composed of relevant data such as age, sex, academic preparation, type of relationship, and annual income.

Conflicts in Romantic Relationships Over FB Use Scale. This instrument was developed by the principal researcher to measure conflicts over FB use in a tridimensional model: Partner FB intrusion, Conflict over FB use, and Jealousy over FB use. For this, the principal researcher originally developed 30 items (10 by dimension) that were submitted to the opinion of eight judges with the objective of identifying whether the items of the instruments were pertinent (Lawshe method). The Content Validity Ratio (CVR_critical_) was used to refuse or withhold the items. To interpret the results, we used the critical values recalculated by Wilson et al. [31]. According to these authors, the minimum value required for eight judges was 0.693 (level of significance for two-tailed test = 0.05) to accept a value as essential. After making the calculation, we identified eight items with values less than 0.693 that were eliminated from the instrument. The 22-item version was rated using a five-point Likert scale: 1 (*Never*), 2 (*Seldom*), 3 (*Sometimes*), 4 (*Usually*), and 5 (*Always*). In this study, the three subscales obtained an internal consistency index of Cronbach’s alpha that ranged between 0.90–0.95.

### 2.4. Data Analysis

In this study, the STATA 15 statistical program was used to perform descriptive statistics (means and typical deviations), data distribution analysis (kurtosis, skewness, Kolmogorov–Smirnov, Shapiro–Wilk), item discrimination index, factor reliability analysis, and correlations among the total scores of the three subscales. Besides, a confirmatory factor analysis with the maximum likelihood estimation method and Satorra–Bentler adjustments were made; these corrections are used when data is not normally distributed [32]. To evaluate the adjustment of the models, we used the following adjustment indexes: Chi-square test (χ^2^), root mean square error of approximation (RMSEA), Tucker–Lewis Index (TLI), Comparative Fit Index (CFI), and Akaike Information Criterion (AIC). RMSEA values less than 0.05 indicate an adequate adjustment of the model [33]. Likewise, CFI and TLI values greater than 0.90 represent an adequate adjustment of the model [33]. AIC is used to examine the parsimony and compare the models; the model with the lower index shows a lower adjustment [34]. Meanwhile, the regression coefficients of each item on its respective factor should exceed 0.50 to be considered adequate [35].

Once the best adjustment model was identified, an item discrimination analysis through item-total correlation was carried out (r_bis_). Those items greater than 0.30 have acceptable discrimination indexes [36]. At the same time, the reliability of the factors was computed using the Cronbach’s alpha coefficient and the omega coefficient; both indexes should be greater than 0.70 [37,38]. In addition, following the recommendations of Fornell and Larcker [39], convergent and discriminant validity was examined through the average variance extracted (AVE). To support convergent validity, the AVE must be equal to or greater than 0.50, with which it is established that more than 50% of the variance of the construct is due to its indicators [40]. Moreover, to determine the discriminant validity for each dimension, the maximum shared variance (MSV) and average shared variance (ASV) should be less than the individual AVE value obtained for each factor. 

Last, an analysis of factorial invariance among two groups (men and women) was carried out. This procedure was performed in three steps. First, a preliminary analysis was carried out where the goodness-of-fit of the same model was examined separately in the two samples under study. The next step in the invariance evaluation required the numbers of factors and the pattern of factorial loads to be the same among all of the groups. This model is a denominated configural model, and it is used as a baseline model in the analysis. Once the goodness-of-fit for the configural model was established, the metric invariance test between the groups was carried out. To compare the models, the changes in the χ2 (which must be non-significant) and in the CFI were taken into consideration. The measurement model is completely invariant if the value found in the ΔCFI is lower than 0.01.

## 3. Results

### 3.1. Descriptive Analysis of the Items

First, means and standard deviations were calculated for each item to analyze the distribution properties of the scale. The means of the items ranged between 2.67–4.42, with standard deviations ranging between 0.81–1.45. The Kolmogorov–Smirnov and Shapiro–Wilk normality tests demonstrated that the data was not normally distributed (*p* < 0.001; see Table 2). Given that the data was not normally distributed, Satorra–Bentler adjustments were used to calculate the adjustment of the structural equation models, since the non-normality of the data changes the estimation errors and the global adjustment of the model [32]. 

### 3.2. Structure Validity

The factor structure of the instrument was examined through confirmatory factor analysis with structural equations using the maximum likelihood estimation method. So that, three competitive models were evaluated: a unifactorial model (M1), where the 22 original items were loaded to one factor, a tridimensional model, where the 22 original items were loaded on its respective factor (M2), and a tridimensional model with six items in each of the factors (M3). The M1 did not show an adequate adjustment to the data (see Table 3). This suggests that the factor structure of the scale is not conformed by a single factor. On the other hand, the M2 showed an adequate adjustment (see Table 3), but some items reflected regression coefficients less than 0.50. For this reason, to achieve greater parsimony in the measurement model, it was decided to retain the six items with the highest regression coefficients in each dimension, considering that these were greater than 0.50. After eliminating items six, 10, 18, and 19, M3 was obtained, as it presented an adequate adjustment (see Table 3), and all of its items reflected regression coefficients greater than 0.50. The regression coefficients ranged between 0.55–0.90 (see Table 4). 

### 3.3. Item Analysis

With the 18 items that made up the M3, the discrimination indexes of the three factors through an item-total correlation index (*r_bis_*) were examined. For the Partner FB intrusion factor, the indexes ranged between 0.51–0.75; for the Conflict over FB use factor, the indexes ranged between 0.76–0.89; and for the Jealousy over FB use factor, the indexes ranged between 0.81–0.90. All of the items obtained discrimination indexes greater than 0.30, as recommended in the literature [36,37]. Table 4 presents the discrimination indexes of the items and the standardized regression coefficients. 

### 3.4. Reliability

Then, Cronbach’s alpha internal consistency indexes and omegas coefficients were calculated for the three factors in the scale (Partner FB intrusion, Conflict over FB use, and Jealousy over FB use). The Cronbach alpha indexes of the factors ranged from 0.87 to 0.95, and the omegas coefficients ranged from 0.88 to 0.95. These indexes exceed the minimum recommended by the literature (0.70) to be considered a reliable instrument [37,38]. 

### 3.5. Convergent and Discriminant Validity

Both discriminant and convergent validity were examined through the average variance extracted (AVE). This indicates the variance explained by the construct in the items. The higher the value of the AVE, the lower the error variance. The AVE values obtained for the factors ranged between 0.55–0.75 (see Table 5). For the AVE to be considered as acceptable, the scores must be equal to or greater than 0.50 [39]. Regarding the discriminant validity, the MSV and the ASV of the factors were lower than the AVE (see Table 5). Furthermore, the relationship between the factors in the scale on its final version (M3) was analyzed through Pearson’s correlation coefficient. The result obtained proved significant positive relationships that ranged between 0.41–0.71 (see Table 5). 

### 3.6. Analysis of Factorial Invariance across Sex

First, the descriptive data of the measures across sex was calculated (see Table 6). Then, the measurement model was estimated independently for women and men; both groups reached adequate adjustment indexes (see Table 7). Then, it proceeded to the restriction factor saturation through the equivalence of structural relationships in the samples (metric invariance). The configural model was used as a baseline to be contrasted with the metric model through χ2 and ΔCFI. The model showed an adequate fit, but the value of ΔCFI was greater than 0.01 (see Table 7). At the same time, the increase of χ2 (Δχ2 = 94.7, *p* < 0.001) was statistically significant, confirming the absence of factorial invariance.

## 4. Discussion

Although several authors have developed instruments that measure variables associated with conflicts in romantic relationships triggered by technology [9,10], neither in Latin America nor Puerto Rico are there instruments in Spanish that evaluate the consequences of the excessive FB use in romantic relationships. For this reason, it is urgent to propose to the Latin American scientific community a valid and reliable instrument to evaluate this phenomenon. To that effect, the main objective of this research was to develop, validate, and examine the psychometric properties of the Conflicts in Romantic Relationships Over FB Use Scale in a sample of Puerto Rican adults. From the results obtained, we can conclude that the instrument has the appropriate psychometric properties to measure conflicts in romantic relationships in three different, but correlated dimensions: Partner FB intrusion, Conflicts over FB use, and Jealousy over FB use. In addition, the obtained reliability indexes suggest, as established in the literature [37,38], that the three subscales have enough internal consistency to be used as a scientific measurement for future research in Puerto Rico and other Spanish-speaking countries. 

In general, the confirmatory factor analysis showed that the data in the hypothesized model presented a satisfactory adjustment and confirmed the tridimensional structure of the instrument, which suggests that it appropriately fits the theoretical conceptualization used by the author to develop the items of the instrument: partner FB intrusion, conflict over FB use, and jealousy over FB use. These three factors should be considered as independent scales that examine different dimensions of conflicts in romantic relationships over FB use. In fact, the moderate correlation between the factors clearly suggests three differentiable variables. The first subscale, Partner FB intrusion, evaluates the frequency with which participants perceive that FB use by their partner interferes in their relationship. That is, it measures the frequency with which their partner accesses FB while they are having dinner, during a conversation, before going to sleep, and during outdoor activities, among others. The scientific literature has consistently associated these behaviors with feelings of insecurity and dissatisfaction in romantic relationships [6,8,9,10,15,24].

On the other hand, the second subscale, Conflict over FB Use, examines the frequency with which participants perceive that FB use generates conflicts in their relationship. Precisely, it assesses perceived discomfort, arguments, rejection, and deterioration in the relationship. There is scientific evidence that confirms that the use of technology, the Internet, social networks, and cell phones at times when couples are trying to share quality time can create conflicts in the relationship, adversely affect communication, and impact their emotional well-being [6,9,10,20,29]. Finally, the third subscale, Jealousy over FB use, examines those behaviors and feelings associated with the jealousy experienced by an individual when his/her partner uses FB. Measuring this variable is particularly important given that research has shown that the excessive use of social networks can predispose jealousy in romantic relationships, and consequently, the decrease of relationship satisfaction and possible breakups may occur [6,8]. 

Regarding the reliability of the scale, indexes higher than the minimum recommended by the scientific literature were obtained in the three subscales [37,38]. This suggests that the final version of the scale is a stable, reproducible, and consistent instrument in the measurement of partner conflicts over FB use. Similarly, the correlations of each item with the total score demonstrate an outstanding internal consistency. This suggests that the items of the final version adequately discriminate and can differentiate people with diverse levels of conflict associated with FB use in the relationship. The findings also provide support for the convergent validity of the scale, given that the average variance extracted, and the standardized factor loadings of the items exceeded the minimum recommended by the literature [39,40]. As to discriminant validity, the results showed that the three factors do not share a substantial amount of variance with each other, and each measures different dimensions. 

As another important theoretical contribution, our results confirmed that the structure of the instrument is not equivalent between men and women. The absence of factorial invariance makes it impossible for comparisons between women and men to be made, since they could generate erroneous or biased interpretations about the differences found, and it is not certain that they are the result of the real differences in the construct or different responses to the items of the instrument [41,42]. That is, women and men do not experience or interpret conflicts over FB use in the same way, nor do they give the same meaning. Three possible explanations for this finding are inferred. First, women are more aware of the negative implications of FB use in their romantic relationship, so they will be more careful when using FB during quality moments that they share with their partner. Second, women have higher expectations than men about sharing quality time, communication patterns, and being present in the relationship [29,43]; that is, they expect more from the relationship, and therefore will be more sensitive to the negative consequences of social network use. The third possible explanation is that men, due to cultural and gender issues, do not recognize that their relationship is vulnerable due to the frequent FB use and ignore the signs that prove these problems (e.g., discussions related to FB use), while women are more intuitive in their emotions. It is necessary that future investigations deepen on this matter.

In practical terms, it was demonstrated that the final version of the Conflicts in Romantic Relationships Over FB Use Scale can be used for the development of new research in the psychology field in the Caribbean. This is a great advancement, given that in Puerto Rico or the Caribbean, there was no instrument to examine this phenomenon. In addition, it would make it easier for couple therapists to perform screening and appraisals to understand how FB use affects relationship well-being. Recent research in Puerto Rico has shown that the use of technology and SNSs negatively impact relationship satisfaction and the mental health of the individuals [20,29]. For this reason, together with the empirical evidence presented in this paper on the negative effects of FB in romantic relationships, the developed instrument is a practical and effective measurement in the research work of behavioral professionals. 

The final version of the instrument consists of 18 items distributed across three subscales (six items in each). The scores must be calculated by adding the six items of each subscale separately to obtain a specific score. Given the independence of the constructs, a measure should not be generated with the sum of the three subscales. The order of the items in the final version was by category; the first six items correspond to the partner FB intrusion subscale, the following six items belong to the conflict over FB use subscale, and the last six items belong to the jealousy over FB use subscale. The possible scores of all subscales range from 6 to 30. 

As with all research, our study has some limitations. First, the sample gathered was a convenience one, so it was not random. Second, it was not possible to establish the reliability of the instrument over time, as it could only be done through its components. Though, the advanced techniques that were used in the study provide empirical strength to our results. Finally, the procedure to collect the data was not standardized, and this may affect the study means and increase the standard error. Despite its limitations, it is worth mentioning the several strengths that this research holds. In the first place, it is the first developed and validated instrument in Puerto Rico and the Caribbean to measure conflicts in romantic relationships over FB use. In fact, there is no instrument in Spanish or English that simultaneously assesses partner FB intrusion, the conflicts over FB use, and the jealousy experienced by people over FB use. In addition, it offers the Spanish-speaking scientific community a reliable and valid instrument that will enrich research aimed at understanding the ways in which couples perceive that frequent FB use impacts their relationships. 

For future research, it is recommended to administer the scale to another sample of participants to perform the cross-validation procedure. It would also be an added value to examine the temporal reliability through the test–retest technique and perform a new confirmatory factor analysis. It is recommended to validate the Conflicts in Romantic Relationships Over FB Use Scale in other Latin American populations to investigate their psychometric properties in diverse national and international contexts. This will allow the comparison of the behavior of the scale in different international contexts and will facilitate studying the FB phenomenon from a multicultural perspective. 

## 5. Conclusions

The present study showed that the Conflicts in Romantic Relationships Over FB Use Scale has appropriate psychometric properties, which implies a high reliability and a solid internal structure of three latent factors. Given this, it is concluded that the instrument is useful to investigate the phenomenon of partner FB intrusion, the conflicts created as a result, and the jealousy experienced by people. It is expected that the developed instrument will be of benefit for its use in the fields of application and research. In the clinical setting, the instrument can be used to identify and prevent problems associated with FB use in romantic relationships, as well as collaborating in the design of future therapeutic interventions.

## Figures and Tables

**Table 1:** Sociodemographic data of the sample.

	*n*	%
**Sex**		
Female	150	50%
Male	150	50%
**Academic Preparation**		
High school or less	46	15.3%
Associate degree/technical	85	28.3%
Bachelor’s degree	113	37.7%
Master’s degree	38	12.7%
Doctoral degree	18	6.0%
**Type of Relationship**		
Marriage	110	36.7%
Cohabiting (free union)	190	63.3%
**Annual Income**		
$0–25,000	196	65.3%
$26,000–50,000	72	24.0%
$51,000–75,000	23	7.7%
$76,000–100,000	4	1.3%
$101,000 or more	5	1.7%

Note: *N* = 300.

**Table 2:** Descriptive and distribution statistics of the items in the final version of the instrument.

Item	*M*	*SD*	Skewness	Kurtosis	Kolmogorov–Smirnov	Shapiro–Wilk
1	3.92	1.01	−0.45	−0.85	0.23	0.85
2	3.54	1.06	−0.20	−0.60	0.21	0.89
3	3.67	1.02	−0.33	−0.55	0.19	0.89
4	4.42	0.81	−1.41	1.63	0.35	0.72
5	4.00	0.95	−0.59	−0.41	0.22	0.85
7	3.59	1.19	−0.33	−0.93	0.19	0.88
8	3.49	1.35	−0.40	−1.04	0.20	0.87
9	3.28	1.25	−0.15	−0.89	0.18	0.90
11	3.63	1.29	−0.55	−0.81	0.20	0.86
12	3.68	1.34	−0.61	−0.83	0.24	0.84
13	3.41	1.42	−0.35	−1.19	0.20	0.86
14	3.28	1.39	−0.22	−1.18	0.17	0.88
15	3.19	1.18	−0.24	−0.51	0.22	0.89
16	3.04	1.26	−0.06	−0.87	0.18	0.91
17	2.99	1.33	−0.04	−1.07	0.16	0.90
20	2.84	1.38	0.13	−1.16	0.14	0.89
21	3.06	1.35	−0.08	−1.12	0.14	0.90
22	3.15	1.45	−0.07	−1.32	0.17	0.88

Note: *M* = Mean; *SD* = Standard Deviation; Skewness standard error = 0.14; Kurtosis standard error = 0.28. Degrees of freedom Kolmogorov–Smirnov and Shapiro–Wilk = 300, all the values *p* < 0.001.

**Table 3:** Goodness-of-fit test for analyzed models.

Model	χ^2^	χ^2^_sb_	*GL*	*RMSEA*	*RMSEA*_sb_	*CFI*	*CFI*_sb_	*TLI*	*TLI*_sb_	*AIC*
M1	1782.76	1590.44	209	0.16	0.15	0.69	0.71	0.66	0.67	17,946.21
M2	645.19	574.82	206	0.08	0.08	0.92	0.92	0.90	0.91	16,814.64
M3	417.56	367.57	132	0.08	0.07	0.94	0.94	0.93	0.93	13,284.36

Note. sb = Satorra–Bentler adjustments; χ^2^ = Chi-square test; χ^2^_sb_= Corrected Chi-square test; *GL* = degrees of freedom; *RMSEA* = root mean square error of approximation; *RMSEA_sb_* = corrected *RMSEA*; *CFI* = Comparative Fit Index; *CFI_sb_* = Corrected *CFI*; *TLI* = Tucker–Lewis Index; *TLI_sb_* = Corrected *TLI*; *AIC* = Akaike Information Criterion; All statistics χ^2^ and χ^2^_sb_ are significant, *p* < 0.001.

**Table 4:** Item discrimination indexes, regression coefficients (*β*) on its respective dimensions, and confidence intervals. FB: Facebook.

Items	*r_bis_*	*β*	*I.C.* 95%
**Partner FB Intrusion**			
1. My partner accesses FB while we are sharing a casual dinner.	0.71	0.76	[0.71, 0.81]
2. My partner uses FB while we are having a conversation.	0.75	0.82	[0.78, 0.86]
3. My partner uses FB when we are sharing time together.	0.69	0.75	[0.67, 0.82]
4. My partner uses FB before going to bed.	0.51	0.55	[0.47, 0.63]
5. My partner uses FB while doing outdoor activities.	0.70	0.74	[0.68, 0.79]
7. My partner access FB if there is a break in our conversation.	0.70	0.78	[0.73, 0.83]
**Conflict over FB Use**			
8. I have spoken to my partner about my discomfort over their excessive FB use.	0.75	0.76	[0.71, 0.81]
9. My partner and I have had discussions due to their recurrent FB use.	0.81	0.82	[0.78, 0.86]
11. The frequency with which my partner uses FB really bothers me.	0.82	0.87	[0.84, 0.91]
12. My partner’s frequent use of FB makes me feel ignored.	0.83	0.89	[0.86, 0.92]
13. I have expressed to my partner that it bothers me when he/she interrupts a conversation to use FB.	0.78	0.80	[0.75, 0.84]
14. My partner’s frequent use of FB is affecting our relationship.	0.82	0.88	[0.85, 0.91]
**Jealousy over FB Use**			
15. I feel jealous due to my partner’s frequent use of FB.	0.83	0.86	[0.83, 0.89]
16. I feel jealous due to my partner’s interaction with other people.	0.82	0.85	[0.81, 0.88]
17. My partner’s frequent use of FB makes me think that he/she will cheat on me.	0.88	0.90	[0.88, 0.93]
20. My partner’s frequent use of FB makes me suspect that he/she is lying to me.	0.85	0.88	[0.84, 0.92]
21. My partner’s FB use has motivated me to verify with whom he/she is interacting on social networks.	0.79	0.81	[0.76, 0.86]
22. I constantly think about what my partner is doing when he/she is in FB for so long.	0.84	0.88	[0.85, 0.91]

Note: *β* = standardized regression coefficients; *p* = significance; *I.C.* 95% = confidence intervals of regression coefficients.

**Table 5:** Means, standard deviations, alphas, omega coefficient, average variance extracted, and correlations.

	*M*	*SD*	*α*	*ω*	*AVE*	*MSV*	*ASV*	1	2	3
1. Partner FB intrusion	23.12	4.77	0.87	0.88	0.55	0.48	0.34	-	0.69 **	0.44 **
2. Conflict over FB use	20.77	6.97	0.93	0.93	0.70	0.59	0.54	0.62 **	-	0.77 **
3. Jealousy over FB use	18.62	7.04	0.95	0.95	0.75	0.59	0.39	0.41 **	0.71 **	-

Note. *M* = Mean; *SD = standard deviation*; α = Cronbach’s alpha coefficient; *ω* = omega coefficient; *ASV* = average variance extracted; *MSV* = maximum shared variance; *ASV* = average shared variance; ** = significant correlations *p* < 0.001. The values on the diagonal represent the correlations between the latent factors, while the values below the diagonal represent the correlations of the direct scores.

**Table 6:** Descriptive data of the measures across sex.

	*N*	*M*	*SD*	*Median*	*Min.*	*Max.*	*Range*
Partner FB intrusion							
Women	150	23.99	5.22	25.00	11	30	19
Men	150	22.25	4.09	21.50	13	30	17
Conflict over FB use							
Women	150	23.27	5.49	24.00	11	30	19
Men	150	18.26	7.39	19.00	6	30	24
Jealousy over FB use							
Women	150	21.47	5.35	21.00	8	30	22
Men	150	15.09	7.10	15.00	6	30	24

Note. *N* = participants; *M* = means; *SD* = standard deviation; *Min.* = minimum; *Max.* = maximum. (*N* = 300).

**Table 7:** Adjustment indexes for compared models in the factorial invariance analysis.

Model	χ^2^	*GL*	*RMSEA*	*CFI*	*TLI*	*AIC*	∆χ^2^	*p*	∆*CFI*
Women	232.40	130	0.07	0.94	0.93	314.397			
Men	252.17	132	0.08	0.95	0.95	330.173			
Configural	566.30	264	0.06	0.924	0.912	722.337			
Metric	661.00	282	0.07	0.905	0.900	781.045	94.7	<0.001	0.019

Note. χ^2^ = Chi-square test; *GL* = degrees of freedom; *RMSEA* = root mean square error of approximation; *CFI* = Comparative Fit Index; *TLI* = Tucker–Lewis Index; *AIC* = Akaike Information Criterion.
